# Effect of melatonin supplementation in combination with neoadjuvant chemotherapy to miR-210 and CD44 expression and clinical response improvement in locally advanced oral squamous cell carcinoma: a randomized controlled trial

**DOI:** 10.1186/s43046-020-0021-0

**Published:** 2020-02-28

**Authors:** Diani Kartini, Akmal Taher, Sonar Soni Panigoro, Rianto Setiabudy, Sri Widia Jusman, Sofia Mubarika Haryana, Murdani Abdullah, Primariadewi Rustamadji, Denni Joko Purwanto, Noorwati Sutandyo, Indrati Suroyo, Budi Harapan Siregar, Haris Maruli, Saleha Sungkar

**Affiliations:** 1 Oncology Division, Department of Surgery, Dr. Cipto Mangunkusumo National Central General Hospital, Faculty of Medicine Universitas Indonesia, Jakarta, 10430 Indonesia; 2 Department of Urology, Cipto Mangunkusumo Hospital, Faculty of Medicine, Universitas Indonesia, Jakarta, Indonesia; 3 Department of Pharmacology and Therapeutics, Dr. Cipto Mangunkusumo National Central General Hospital, Faculty of Medicine Universitas Indonesia, Jakarta, Indonesia; 4 Department of Biochemistry and Molecular Genetics, Dr. Cipto Mangunkusumo National Central General Hospital, Faculty of Medicine Universitas Indonesia, Jakarta, Indonesia; 5 Department of Histology and Cell Biology, Faculty of Medicine, Public Health and Nursing, Universitas Gadjah Mada, Yogyakarta, Indonesia; 6 Division of Gastroenterology and Hepatology, Department of Internal Medicine, Dr. Cipto Mangunkusumo National Central General Hospital, Jakarta, Indonesia; 7 Department of Pathological Anatomy, Cipto Mangunkusumo Hospital, Faculty of Medicine, Universitas Indonesia, Jakarta, Indonesia; 8 Department of Surgical Oncology, Dharmais Hospital, National Cancer Center, Jakarta, Indonesia; 9 Department of Hematology and Medical Oncology, Dharmais Hospital, National Cancer Center, Jakarta, Indonesia; 10 Department of Radiology, Dr. Cipto Mangunkusumo National Central General Hospital, Jakarta, Indonesia; 11 Oncology Division, Department of Surgery, Persahabatan General Hospital, Jakarta, Indonesia; 12 Department of Parasitology, Dr. Cipto Mangunkusumo National Central General Hospital, Faculty of Medicine Universitas Indonesia, Jakarta, Indonesia

**Keywords:** CD44, Clinical Response, Melatonin, OSCC, Tumor residue percentage

## Abstract

**Background:**

Squamous cell carcinoma of the oral cavity (OSCC) is the sixth most common malignancy. Surgery is mainstay treatment for oral cancers. Surgery in locally advanced OSCC presents many challenges primarily because the head and neck have critical structures that can be damaged by tumor or treatment. It is thought that neoadjuvant chemotherapy (NC) in locally advanced OSCC is able to shrink tumor size. Chemoresistancy is a problem due to hypoxic microenvironment characterized by increased expression of HIF-1α. It is also regulated by miR-210 as well as increased expression of CD44 and CD133. Melatonin has a powerful antioxidant and oncostatic effects that are expected to improve tumor hypoxia and clinical response. Fifty patients with OSCC were included and randomized. miR-210 and CD44 expression were measured before and after intervention using qRT-PCR absolute quantification, and clinical response was evaluated according to RECIST 1.1 criteria. This study aims to determine the effect of melatonin in improving the clinical response of patients with locally advanced oral squamous cell carcinoma (OSCC) after neoadjuvant chemotherapy to miR-210 and CD44 expression.

**Results:**

Melatonin administration reduced miR-210 levels but not significant (*p* = 0.767). CD44 expression also decreased in the melatonin group compared with placebo yet was not significant (*p* = 0.103). There was a decrease in the expression of miR-210 and CD44 followed by a decrease in the percentage of residual tumor but not significant (*p* = 0.114).

**Conclusion:**

In OSCC, the addition of 20-mg melatonin to neoadjuvant chemotherapy (NC) reduced the expression of miR-210 and CD44 and decreased the percentage of tumor residue; however, no statistically significant result was observed.

**Trial registration:**

This study is registered to ClinicalTrials.gov under trial registration number: NCT04137627 with date of registration on October 22, 2019—retrospectively registered, accessible from: https://clinicaltrials.gov/ct2/show/NCT04137627

## Background

Oral cavity carcinoma is the 6th most commonly found malignancy in the world [1]. Most of these oral cavity carcinoma (OCC) are often found in developing countries [2]. The most commonly observed histological subtype of oral cavity carcinoma is squamous cell carcinoma, which can be found in the mucosa of the oral cavity, gingiva, hard palate, tongue, and lips [1]. The mainstay therapy for oral squamous cell carcinoma (OSCC) is surgery; however, it is challenging due to the complexity of the head and neck anatomical structure and the change in oral cavity anatomy due to cancer growth. Neoadjuvant chemotherapy (NC) is administered on oral squamous cell carcinoma (OSCC) to reduce the size of the tumor; therefore, surgery can be performed easier. Surgery for oral squamous cell carcinoma can improve the 5-year survival rate by 45% at T4b stage [3].

An issue that is often encountered in oral squamous cell carcinoma (OSCC) therapy is chemoresistance. Mechanisms underlying tumor resistance to anti-tumor drugs involve several factors, namely pharmacokinetic resistance, intrinsic tumor cells resistance, and factors associated with tumor microenvironment [4, 5]. This hypoxic microenvironment condition is a factor associated with processes related to energy metabolism, cell proliferation and survival, angiogenesis, adhesion, and motility. The imbalance of oxygen supply and consumption causes the disruption of transportation and distribution of chemotherapy in blood vessels [6]. Hypoxia also worsens blood vessel disorganization caused by the increased production of angiogenic factors by tumor cells and stroma [7]. Hypoxic conditions will also cause an increase in the expression of reactive oxygen species (ROS), which results in chemoresistance. Lee Dj et al. mentioned in their study that ROS has a dual role as a mediator of angiogenesis and metastatic signals at sub-optimal concentrations but suppresses angiogenesis and tumor cell growth at low concentrations [8].

In addition to the above mechanism, chemoresistance can be caused by cancer stem cells that are dormant and have a slow kinetic cell cycle; thus, making them more likely to escape chemotherapy [9]. Cancer stem cells originating from the head and neck can be identified based on CD44+ expression. These cells are shown to have the ability for self-renewal, differentiation, clonogenicity, and are resistant to cytostatic cisplatin and gemcitabine [10, 11]. A study by Prince et al. suggests the ability of CD44+ to initiate tumorigenesis in mice. Cells which possess high levels of CD44 expression is shown to renew a heterogeneous tumor phenotype, but do not occur in cells with low CD44 expression [11]. These markers are often found and targeted for cancer treatment. Therefore, if there is an increase in CD44 it can cause chemoresistance.

The function of cancer stem cells can be modulated by miRNA by influencing the level of expression of target genes and proteins involved in the signaling pathway of cell proliferation and death [12]. Aside from cancer stem cells, chemoresistance is also regulated by miRNA, which is non-coding RNA [13]. miRNA plays a role in regulating cancer stem cells by making them to possess properties of self-renewal, pluripotency, and neoplastic [12]. There are several types of miRNA associated with hypoxic conditions [14]. miR-210 is one of the several markers that is considered to have an important role and held the largest representation in hypoxic conditions [15, 16]. Hence, it is often referred as *hypoxamir* due to its high and consistent expression in hypoxic condition in various types of cells [16].

The state of tumor resistance to chemoresistance requires the use of antioxidants to overcome the hypoxic state which can be achieved by using melatonin. Melatonin also acts as an oncostatic by inducing apoptosis of cancer cells. Melatonin and its metabolite derivates are known as antioxidants and potent radical scavenger since it can eliminate 10 ROS due to its ability to form antioxidant cascade; thus, it is far more effective than other antioxidants [17].

The administration of melatonin is expected to improve the state of tumor hypoxia; hence, it can improve clinical response by reducing the percentage of tumor residues. Nevertheless, there has not been any experimental study which investigates the effect of melatonin in improving tumor clinical response to chemotherapy associated with the expression of biomarkers such as miR-210 and CD44, especially in patients with locally advanced oral squamous cell carcinoma (OSCC). This study is aimed to determine the effect of melatonin in improving the clinical response of patients with locally advanced oral squamous cell carcinoma (OSCC) after neoadjuvant chemotherapy to miR-210 and CD44 expression.

## Methods

### Study design and subjects

**Fig. 1:**
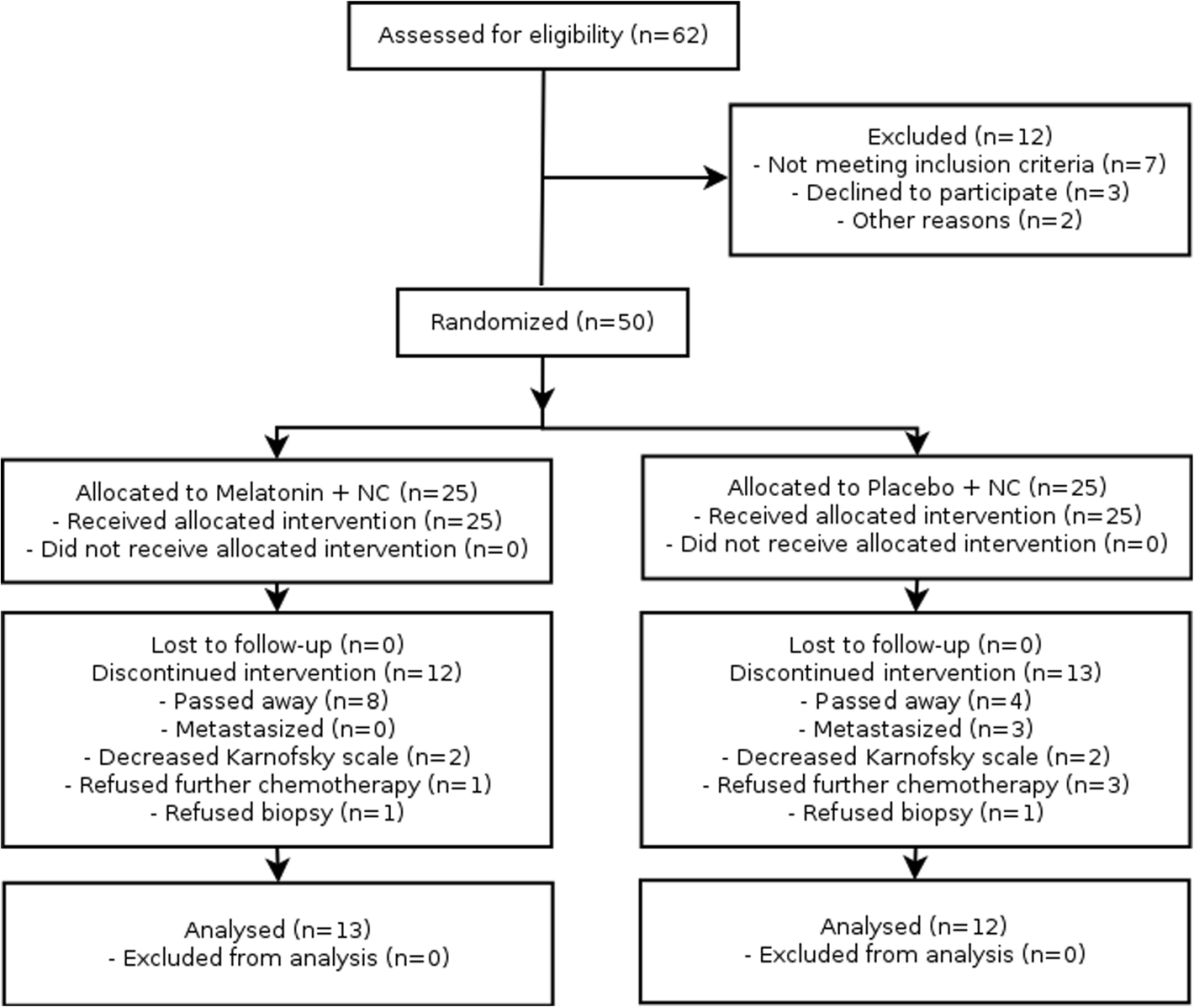
Flow of research subjects enrollment

This study is a double blinded, parallel, randomized controlled trial with placebo as a comparison conducted in June 2017 to July 2018. Our study populations were gathered from Cipto Mangunkusumo Hospital (48 patients) and Dharmais Hospital, National Cancer Center, Indonesia (2 patients). The sampling technique used in this study was consecutive sampling with randomization. Randomization of patient was done by a third party using computerized block randomization; concealment was done by giving serial number on drug preparations. The study was conducted in compliance with the rules of Good Clinical Practice (GCP) and it was approved by the Institutional Review Board (IRB), and all subjects provided informed consents. The study reporting adheres to the CONSORT guidelines for reporting clinical trials. Inclusion criteria are oral squamous cell carcinoma stage IVA and IVB patients who would be treated with neoadjuvant chemotherapy (NC), and the patient had never undergone definitive surgery or had never been treated with neoadjuvant chemotherapy. Of the 50 randomized oral squamous cell carcinoma patients, only half of all patients completed the study protocol (13 patients in the melatonin group and 12 patients in the placebo group). Flow of research subjects is described in Fig. 1.

### Study intervention

Patients who will be given intervention will have an open biopsy in advance (incisional biopsy) or core biopsy which is used to confirm the histopathological characteristics of locally advanced oral squamous cell carcinoma (OSCC) and obtain patient samples before receiving treatment. Fifty randomized patients will be divided into two groups: 25 patients receive a combination of melatonin and neoadjuvant chemotherapy (NC) (taxane, cisplatin, and 5-fluorouracil) for 3 cycles and 25 patients will receive neoadjuvant chemotherapy alone. Neoadjuvant chemotherapy will be fulfilled in 3 cycles; each cycle has an interval of 3 weeks. Seven days prior to neoadjuvant chemotherapy (NC), the patient was administered with 20 mg of melatonin or placebo and continued to be consumed in conjunction with neoadjuvant chemotherapy (NC) for up to 3 cycles.

### Clinical response evaluation

To assess clinical response, this study used the RECIST 1.1 criteria, which consists of complete response (CR), partial response (PR), progressive disease (PD), and stable disease (SD). MRI will be performed to assess clinical response before and after the administration of neoadjuvant chemotherapy. Surgery will be performed on CR and PR patients, followed by tissue and blood examination with qRT-PCR. Meanwhile, secondary biopsy will be performed in patients with SD and PD, followed by blood examination with qRT-PCR.

### Examination of gene expression

CD44 and miR-210 gene amplification process primer design was performed using the PrimerQuest Tool IDT. The sequence information for each gene was obtained from the National Center for Biotechnology Information (NCBI) database. In addition, gBlocks Gene Fragments IDT (integrated DNA technologies) are used as sequence selection for each gene and gBlocks synthesis. Changes in gene expression concentration were assessed using the absolute quantification qRT-PCR method. There are several stages of the examination of gene expression consisting of RNA isolation, cDNA synthesis, and qRT-PCR two-step absolute quantification. The concentration of RNA level gene expression obtained from qPCR will be analyzed based on the positive control standard curves of each gene. Positive control of each gene was the oligosynthetics of CD44 and miR-210. Negative control was technical PCR negative control, namely no template control.

### Statistical analysis

Statistical analysis was performed using IBM© SPSS® version 20. Sample size was calculated on the basis of historical trial [18], and the incidence of locally advanced OSCC at the participating institute. Using a formula for superiority trial design with continuous variable, a sample size of 42 patients was needed to achieve the objectives of this study assuming 5% level of significance and 80% power. Assuming a 10% possibility of drop out; hence, a total of 46 patients were required for this study. Normality of data distribution was tested using the Saphiro-Wilk test. Data with normal distribution is shown in mean (SD), while data with non-normal distribution is shown in median (range). The difference between the treatment group and control group is analyzed with the Saphiro-Wilk normality test. For normal distribution data will be tested using independent *t* test, while for non-normal distribution data will be tested using the Mann-Whitney test. Changes with *p* values of less than 0.05 were considered statistically significant.

## Results

In this study, 50 subjects were enrolled. However, during the 1-year follow-up period, only 25 patients completed the study due to the clinical condition of the patients and exclusion to the patients.

Study result showed decreased expression of miR-210 in both groups receiving melatonin (*p* < 0.001) and placebo (*p* < 0.001). There was a 67.1% decrease in miRNA-210 concentration after melatonin administration, compared with placebo group which showed a 59.2% decrease in miRNA-210 concentration. However, the difference in the reduction of miR-210 expression between the melatonin and placebo groups was not significantly different (*p* = 0.767) (Table 1).

**Table 1:** Concentration of miR-210 expression before and after melatonin and placebo administration

miRNA-210	Mean (SD)		
	Melatonin (*n* = 13)	Placebo (*n* = 12)	*p*
Before	162.8 (54.92)	175.2 (34.30)	0.767
After	53.8 (16.65)	53.5 (14.28)	
Difference (after − before)	− 109.09 (51.62)	− 103.71 (36.24)	

**Fig. 2:**
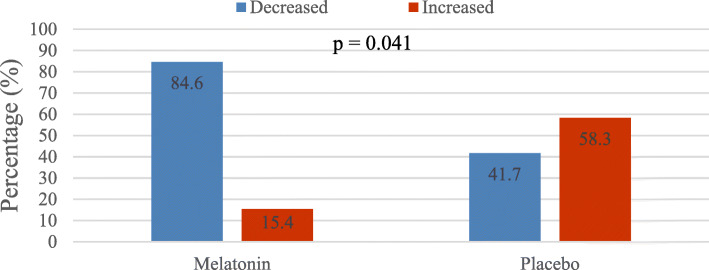
Percentage of subjects showing change in CD44 expression

The administration of 20-mg melatonin per day reduced CD44 expression in 11 out of 13 subjects (*p* = 0.041), and the degree of reduction is two-fold greater compared with subjects receiving placebo (RR = 2.03). On the contrary, in the placebo group, 7 out of 12 subjects experienced an increase in CD44 expression (Fig. 2). In subjects receiving melatonin, there was a 32.67% reduction in CD44 concentration after melatonin administration. Meanwhile, there was an 86.32% increase in CD44 concentration in subject receiving placebo. Nevertheless, considering the degree of reduction of CD44 expression, there were no significant differences between the two groups (*p* = 0.103) (Table 2).

**Table 2:** Concentration of CD44 expression before and after melatonin and placebo administration

CD44	Median (range)		
	Melatonin (*n* = 13)	Placebo (*n* = 12)	*p*
Before	0.0349 (0.0048–0.1440)	0.0095 (0.0002–0.1170)	0.103
After	0.0115 (0.0003–0.2040)	0.0187 (0.0012–0.2780)	
Difference (after − before)	− 0.0114 (− 0.0994 **→** + 0.1736)	0.0082 (− 0.1109 **→** + 0.2773)	

### The effect of melatonin administration on biomarker concentration and tumor residue percentage

In this study, clinical response was evaluated using RECIST 1.1 pre- and post administration of neoadjuvant chemotherapy. The results obtained are complete response, partial response, stable disease, and progressive disease. Following that, to obtain clinical response in numerical value, the percentage of tumor residue was calculated by considering the size of the tumor before giving neoadjuvant chemotherapy as 100% then adding or subtracting the tumor percentage from RECIST calculation.

In the melatonin group, it can be seen that the decrease in CD44 is followed by a decrease in the percentage of tumor residue to 84.22%, but when the CD44 expression is increased, the percentage of tumor residue increases as well. The decrease in miR-210 expression is also followed by a decrease in the percentage of remaining tumors to be 82.74% (Table 3).

**Table 3:** Effect of melatonin to biomarker concentration and tumor residue percentage

Variable	Melatonin (*n* = 13)	%Tumor residue	*p*
miRNA-210			
Decreased	13	82.74 (37.5)	–
Increased	0	–	
CD 44			
Decreased	11	84.22	0.114
Increased	2	115.06	

## Discussion

In this study, the decreased expression of miR-210 was observed in both study groups. This finding was significant (*p* < 0.001). The degree of reduction was greater in the melatonin group. However, the difference in reduction between the two groups was not significant (*p* = 0.767). The role of melatonin as an antioxidant and radical scavenger is shown in its effect on the nature of stem cell or through a decrease in ROS that is followed by the reduction of miR-210 [19]. A study by Kim et al. reported the increased expression of miR-210 with a 24-h peak in adipose stem cells that had been put in a hypoxic condition for 6 h. This increase can be inhibited by a ROS scavenger, namely *N*-acetylcysteine (NAC) [20]. The finding suggests that miR-210 regulation is mediated by ROS especially in hypoxic state.

The chemotherapy administration in the placebo group was thought to play a role in the decreased expression of miRNA. The expression of miRNA through pri-MRNA transcription modulation can be regulated through the DNA damage response (DDR) pathway occurring after the administration of cisplatin. A decrease in miR-210 expression was observed in a study regarding the ability of 5-FU to modify miRNA in C22.20 and HC.21 cell cultures of colon carcinoma [21]. Another study conducted by Shah et al. elucidated that the decreased expression of miR-210 could be due to an error in miRNA expression owing to the entry of 5-FU metabolites (FdUTP and FUTP) into the gene transcript miRNA or the production of fluorinated mature miRNA; thus, modifying its main function [22].

In this study, a reduction of tumor residue percentage was observed following the decreased expression of miR-210 and CD44, though it was insignificant. It is thought that the melatonin administration caused the decreased expression of miR-210 due to the reduced expression of ROS through PDGFR-β, Akt, ERK1/2, NF-κB, and Elk1 pathways along with the effect of chemotherapy to alteration in miRNA transcription, increase in apoptosis, inhibition of invasion, and reduction in cell proliferation. These chain of events may contribute to the reduction of tumor residue percentage [20].

Decreased CD44 expression was observed in 11 subjects (84.6%) in the melatonin group and 5 subjects (41.7%) in the placebo group, although it was not significant when compared between the two groups (*p* = 0.103). The role of melatonin in the inhibition of tumor growth and metastasis is through the activation of cell signaling pathways which depends on the cancer cell type and the dose/concentration of melatonin itself [22, 23]. Goncalves et al. reported the administration of 1-mM melatonin weakened stemness property in cancer stem cells on breast cancer in both cell cultures (CMT-U229 and MCF-7) with CD44^+^/CD24^−^ cell phenotype [24]. Epithelial mesenchymal transition (EMT) is a marker for invasiveness which consists of OCT-4, E-cadherin, N-cadherin, and Vimentin.

There have been reports of the insignificant reduction of CD44 expression in contrary to a study by Goncalves, Kocak, and Akbarzadeh, presumably due to the use of cell culture instead of a tissue sample, thus permitting better control to confounding factors and requiring different examination techniques of CD44 [24–26]. A study by Ullman mentioned a link between miR-210 and regulation of CD44; thus, the reduction of CD44 in this study may come as a result from the decrease of miR-210 [27]. Increased miR-210 expression correlated with E-cadherin expression regulation that was caused by hypoxic microenvironment along the tumor; thus, increasing migration, invasion, and proliferation in vitro and in vivo [28]. In turn, the increase of miR-210 expression would also trigger the enhancement of CD44 expression in hypoxic state [27]. miR-210 targeted iron-sulfur cluster assembly protein (ISCU) in a state of hypoxia as a part of metabolic response. The enhancement of miR-210 expression accompanied by ISCU inhibition would cause the reduction in the tricarbolate acid cycle activity and an increase in lactate production; thus, giving rise to cell proliferation [27].

Because this study was done in vitro, it has several limitations, namely difficulty in interpreting the association of in vitro phenotype to in vivo outcome, mainly in the interest of toxic chemotherapeutic drugs which possess very narrow therapeutic index [29]. The effect of drugs on cell culture/cell line could differ to that of clinical effect due to the fact that clinical effect is influenced by cellular or tissue microenvironment and the pharmacokinetic of the drug [30, 31]. Furthermore, melatonin’s low bioavailability also affects its clinical response and the expression of miR-210 and CD44. This is due to the melatonin’s inability to be absorbed entirely and could undergo first-pass metabolism. Also, melatonin’s bioavailability may vary between subjects; therefore, its clinical effect may not be apparent.

Based on the discussion above, it can be concluded that the administration of 20-mg melatonin per day can reduce the expression of miR210 (*p* = 0.767) and CD44 (*p* = 0.103), though both were not statistically significant. The clinical response of the melatonin administration was also not significant in both groups. This statistical insignificance may be due to the limited number of subjects who finished the protocol. Moreover, this study was conducted in tumor tissue sample; thus, leaving confounding factor to be uncontrolled.

## Conclusion

The present study suggests that in oral squamous cell carcinoma (OSCC), the addition of 20-mg melatonin to neoadjuvant chemotherapy (NC) (docetaxel, cisplatin, and 5-FU) reduced the expression of miR-210 and CD44 and decreased the percentage of tumor residues; however, no statistically significant result was observed.

## Data Availability

The datasets generated and/or analyzed during the current study are not publicly available due to the institutional policy to protect subject’s confidentiality, but are available from the corresponding author on reasonable request.

## Abbreviations

5-FU: 5-Fluorouracil
CR: Complete response
DDR: DNA damage response
EMT: Epithelial mesenchymal transition
ISCU: Iron-sulfur cluster assembly protein
NAC: *N*-acetylcysteine
NC: Neoadjuvant chemotherapy
OSCC: Oral squamous cell carcinoma
PD: Progressive disease
PR: Partial response
ROS: Reactive oxygen species
SD: Stable disease

## References

[1] Warnakulasuriya S. Global epidemiology of oral and oropharyngeal cancer. Oral Oncol. 2009;45(4-5):309–16. 18804401 10.1016/j.oraloncology.2008.06.002

[2] Szafarowski T, Szczepanski MJ. Cancer stem cells in head and neck squamous cell carcinoma. Otolaryngol Pol. 2014;68(3):105–11. 24837904 10.1016/j.otpol.2013.10.010

[3] Liao CT, Chang JT, Wang HM, Ng SH, Hsueh C, Lee LY, et al. Surgical outcome of T4a and resected T4b oral cavity cancer. Cancer. 2006;107(2):337–44. 16770782 10.1002/cncr.21984

[4] Gatti L, Zunino F. Overview of tumor cell chemoresistance mechanisms. Methods Mol Med. 2005;111:127–48. 15911977 10.1385/1-59259-889-7:127

[5] Gottesman MM. Mechanisms of cancer drug resistance. Annu Rev Med. 2002;53(1):615–27. 11818492 10.1146/annurev.med.53.082901.103929

[6] Li JZ, Gao W, Chan JY, Ho WK, Wong TS. Hypoxia in head and neck squamous cell carcinoma. ISRN Otolaryngol. 2012 Oct 16;2012:708974. 23762617 10.5402/2012/708974 PMC3671689

[7] Carmeliet P, Jain RK. Principles and mechanisms of vessel normalization for cancer and other angiogenic diseases. Nat Rev Drug Discov. 2011;10(6):417–27. 21629292 10.1038/nrd3455

[8] Lee DJ, Kang SW. Reactive oxygen species and tumor metastasis. Mol Cells. 2013;35(2):93–8 (Retracted article). 23456330 10.1007/s10059-013-0034-9 PMC3887897

[9] Kusumbe AP, Bapat SA. Cancer stem cells and aneuploid populations within developing tumors are the major determinants of tumor dormancy. Cancer Res. 2009;69(24):9245–53. 19951996 10.1158/0008-5472.CAN-09-2802

[10] Shanbhag VL. Understanding cancer stem cells in head and neck cancer: an insight from oral medicine point of view. Oncobiol Targets. 2015;2:24–8.

[11] Prince ME, Sivanandan R, Kaczorowski A, Wolf GT, Kaplan MJ, Dalerba P, et al. Identification of a subpopulation of cells with cancer stem cell properties in head and neck squamous cell carcinoma. Proc Natl Acad Sci. 2007;104(3):973–8. 17210912 10.1073/pnas.0610117104 PMC1783424

[12] Garg M. Potential therapeutic applications of microRNAs in response to DNA damage in cancer stem cells.J. Stem Cells. 2011;6(2):51–65. 22997846

[13] Huppi K, Volfovsky N, Mackiewicz M, Runfola T, Jones TL, Martin SE, et al. MicroRNAs and genomic instability. Semin Cancer Biol. 2007;17(1):65–73. 17113784 10.1016/j.semcancer.2006.10.004 PMC1839944

[14] Kulshreshtha R, Ferracin M, Negrini M, Calin GA, Davuluri RV, Ivan M. Regulation of microRNA expression: the hypoxic component. Cell Cycle. 2007;6(12):1426–31. 17582223

[15] Gee HE, Camps C, Buffa FM, Patiar S, Winter SC, Betts G, et al. Hsa-Mir-210 is a marker of tumor hypoxia and a prognostic factor in head and neck cancer. Cancer. 2010;116(9):2148–58. 20187102 10.1002/cncr.25009

[16] Chan YC, Banerjee J, Choi SY, Sen CK. miR-210: The master hypoxamir. Microcirculation. 2012;19(3):215–23. 22171547 10.1111/j.1549-8719.2011.00154.x PMC3399423

[17] Tan DX, Manchester LC, Esteban-Zubero E, Zhou Z, Reiter RJ. Melatonin as a potent and inducible endogenous antioxidant: synthesis and metabolism. Molecules. 2015;20(10):18886–906. 26501252 10.3390/molecules201018886 PMC6332205

[18] Lin PY, Yu CH, Wang JT, Chen HH, Cheng SJ, Kuo MY, Chiang CP. Expression of hypoxia-inducible factor-1 alpha is significantly associated with the progression and prognosis of oral squamous cell carcinomas in Taiwan. J Oral Pathol Med. 2008;37(1):18–25. 18154573 10.1111/j.1600-0714.2007.00571.x

[19] de Almeida Chuffa LG, Fioruci BA, Seiva FRF. Melatonin and their protective role on oxidative cell damage: interplay between oxidative stress and tumorigenesis. In: Castroviejo DA, Rusanova I, Escames G, editors. New Developments in Melatonin Research: Spain, Nova Publishers; 2013. p. 121–38.

[20] Kim JH, Park SG, Song SY, Kim JK, Sung JH. Reactive oxygen species responsive miR-210 regulates proliferation and migration of adipose derived stem cells via PTPN2. Cell Death Dis. 2013;4:e588. 23579275 10.1038/cddis.2013.117 PMC3641340

[21] Rossi L, Bonmassar E, Faraoni I. Modification of miR gene expression pattern in human colon cancer cells following exposure to 5-fluorouracil in vitro. Pharmacol Res. 2007;56(3):248–53. 17702597 10.1016/j.phrs.2007.07.001

[22] Shah MY, Pan X, Fix LN, Farwell MA, Zhang B. 5-fluorouracil drug alters the microrna expression profiles in MCF-7 breast cancer cells. J Cell Physiol. 2011;226(7):1868–78. 21506117 10.1002/jcp.22517

[23] Jung B, Ahmad N. Melatonin in cancer management: progress and promise. Cancer Res. 2006;66(20):9789–93. 17047036 10.1158/0008-5472.CAN-06-1776

[24] Gonçalves N, Colombo J, Lopes JR, Gelaleti GB, Moschetta MG, Sonehara NM, et al. Effect of melatonin in epithelial mesenchymal transition markers and invasive properties of breast cancer stem cells of canine and human cell lines. PLoS One. 2016;11(3):e0150407. 26934679 10.1371/journal.pone.0150407 PMC4774906

[25] Koçak N, Dönmez H, Yildirim İH. Effects of melatonin on apoptosis and cell differentiation in MCF-7 derived cancer stem cells. Cell Mol Biol (Noisy-le-grand). 2018;64(12):56–61. 30301504

[26] Akbarzadeh M, Movassaghpour AA, Ghanbari H, Kheirandish M, Maroufi NZ, Rahbarghazi R, et al. c. Sci Rep. 2017; 7: 17062. 10.1038/s41598-017-16940-y PMC5719004 29213108

[27] Ullmann P, Qureshi-Baig K, Rodriguez F, Ginolhac A, Nonnenmacher Y, Ternes D, et al. Hypoxia-responsive miR210 promotes self-renewal capacity of colon tumor-initiating cells by repressing ISCU and by inducing lactate production. Oncotarget. 2016;7(40):65455–70. 10.18632/oncotarget.11772 PMC5323168 27589845

[28] Tang T, Yang Z, Zhu Q, Wu Y, Sun K, Alahdal M, et al. Up-regulation of miR-210 induced by a hypoxic microenvironment promotes breast cancer stem cell metastasis, proliferation, and self-renewal by targeting E-cadherin. FASEB J. 2018:fj201801013R. 10.1096/fj.201801013R 30188754

[29] Welsh M, Mangravite L, Medina MW, Tantisira K, Zhang W, Huang RS, et al. Pharmacogenomic discovery using cell-based models. Pharmacol Rev. 2009;61(4):413–29. 20038569 10.1124/pr.109.001461 PMC2802425

[30] Voskoglou-Nomikos T, Pater JL, Seymour L. Clinical predictive value of the in vitro cell line, human xenograft, and mouse allograft preclinical cancer models. Clin Cancer Res. 2003;9(11):4227–39. 14519650

[31] Zeeberg BR, Kohn KW, Kahn A, Larionov V, Weinstein JN, Reinhold W, et al. Concordance of gene expression and functional correlation patterns across the NCI-60 cell lines and the Cancer Genome Atlas glioblastoma samples. PLoS One. 2012;7(7):e40062. 22848369 10.1371/journal.pone.0040062 PMC3406063

